# Supplementary material for: Variability in Skeletal Muscle Protein Synthesis Rates in Critically Ill Patients
Source: Nutrients. 2022 Sep 10;14(18):3733. doi: 10.3390/nu14183733 (PMC9501828; doi:10.3390/nu14183733)

Supplementary material to:

Variability in skeletal muscle protein synthesis rates in critically ill patients.

Inga Tjäder, Maria Klaude, Ali Ait Hssain, Christelle Guillet, Inger Nennesmo, Jan Wernerman and Olav Rooyackers

Content:

Table S1. Patient Characteristics

Table S2. Enrichments

Table S1 Patients characteristics

Pat.	Diagnosis	BMI (kg/m^2)	Days in ICU at study	Length of ICU stay	APACHE II	SOFA at admission	SOFA at study	Parenteral nutrition	Enteral nutrition	Antibiotics	Actrapid infusion	Inotropic support	Cortison	Survival
1	Abdominal aortic aneurysm, pneumonia	32	2	5	12	7	7	no	yes	s	yes, int	t	subst	survived
2	Pneumonia	28	1	13	19	7	9	no	no	t	yes	s	no	survived
3	Stroke, pneumonia, cardiac arrest	27	42	81	19	7	8	yes	yes	t/+f	yes	no	subst	died at day 82 in hospital
4	Respiratory failure after surgery	25	1	9	22	5	5	no	yes	d	yes	t	subst	survived
5	Sepsis	31	2	3	27	3	1	no	yes	d	yes	s	subst	survived
6	Respiratory failure, COPD	29	1	7	30	5	6	yes	yes	s	yes	d	yes	survived
7	Respiratory failure after surgery	33	2	19	25	4	2	yes	yes	d	yes	no	no	survived
8	Esofageal resection, COPD	24	7	29	21	8	5	yes	no	s	yes, int	s	no	survived
9	Respiratory failure, COPD	22	1	6	21	7	6	no	yes	s	yes	s	yes	survived
10	Pneumonia, COPD	20	3	8	39	8	5	no	no	s	yes	s	yes	survived
11	Abdominal aortic aneurysm	27	35	91	16	11	4	yes	yes	t/+f	yes	t	subst	survived
12	Multiple ribfractures, respiratory failure	28	6	16	19	5	9	yes	yes	d	yes	d	no	died at day 95 in hospital
13	Respiratory failure and AMI	25	2	4	21	4	4	yes	yes	s	yes	s	yes	died at day 16 in ICU
14	Pneumonia	22	2	6	30	8	7	yes	no	s	yes	s	no	survived
15	Respiratory failure	28	2	69	14	8	7	yes	no	s/+f	no	no	no	survived
16	Abdominal sepsis after surgery, COPD	42	1	8	29	12	12	yes	no	s	yes	d	subst	survived
17	Abdominal sepsis after surgery	50	6	21	13	7	3	yes	yes	t/+f	yes	s	no	died at day 13 in hospital
														survived
**Mean**		**29**			**22**	**7**	**6**	**11 / 6**	**10 / 7**		**16 / 1**			
**± SD**		**7**			**7**	**2**	**3**							

Abbreviations; COPD: chronic obstructive pulmonary disease; AMI: acute myocardial infarction; BMI: body mass index; APACHE: acute physiology and chronic health evaluation; SOFA: sequential organ failure assessment; M: male; F: female; s/d/t/+f denote single/double/triple therapy; /+f denotes antifungal therapy; int. denotes intermittent; subst.denotes substitution therapy.

Table S2 Enrichments

Enrichments (Molar Percent Excess; MPE) of D5-phenylalanine in the precursor pool (plasma) and the different protein pools from skeletal muscle of the 17 critically patients. Data on the mitochondrial protein fraction is published in: *Fredriksson K, Tjäder I, Keller P, Petrovic A, Ahlman B, Schéele C, Wernerman J, Timmons JA, Rooyackers O (2008). Dysregulation of mitochondrial dynamics and the muscle transcriptome in ICU patients suffering from sepsis induced multiple organ failure. PlosOne 3(11): e3686.*

Patient	Precursor (AUC, MPE)	MPE mixed protein	MPE mito protein	MPE myosin	MPE actin	FSR mixed protein	FRS mito protein	FSR myosin	FSR actin
1	569	0.0059	0.00958	0.00311	0.01240	1.49	2.43	0.79	3.14
2	500	0.0058	0.00860	0.01737	0.01172	1.67	2.48	5.00	3.37
3	1095	0.0819	0.08863	0.05284	0.04852	10.77	11.65	6.95	6.38
4	1096	0.0131	0.01913	0.00678	0.02298	1.72	2.51	0.89	3.02
5	1132	0.0112	0.01250	0.00558	0.01975	1.42	1.59	0.71	2.51
6	1023	0.0124	0.01414	0.00568	0.02250	1.74	1.99	0.80	3.17
7	1124	0.0145	0.01298	0.00716	0.02495	1.85	1.66	0.92	3.20
8	991	0.0176	0.01765	0.00601	0.02584	2.56	2.56	0.87	3.75
9	1268	0.0234	0.02915	0.00920	0.02133	2.65	3.31	1.04	2.42
10	1219	0.0217	0.02943	0.00402	0.02423	2.56	3.48	0.48	2.86
11	1109	0.0402	0.04876	0.00993	0.03408	5.21	6.33	1.29	4.42
12	1152	0.0215	0.02580	0.00364	0.02810	2.68	3.23	0.45	3.51
13	1107	0.0134	0.01932	0.00338	0.02118	1.74	2.51	0.44	2.76
14	1072	0.0125	0.01523	0.00284	0.01175	1.68	2.05	0.38	1.58
15	1037	0.0114	0.01398	0.00221	0.01664	1.58	1.94	0.31	2.31
16	1036	0.0105	0.01119	0.00304	0.01407	1.46	1.56	0.42	1.96
17	1043	0.0241	0.02352	0.00465	0.02632	3.33	3.25	0.64	3.63

Table S3 Morphological changes

Slides of both the right and the left leg of the critically ill patients were scored for degeneration (1 if present), atrophy (1 if present), central nuclei (1 if present) and infiltration of inflammatory cells (1 if present).

	Degeneration		Atrophy		Central nuclei		Infiltrating cells	
Patient	**right**	**left**	**right**	**left**	**right**	**left**	**right**	**left**
**1**								
**2**			1	1				
**3**	1	1			1	1		
**4**								
**5**								
**6**			1	1	1	1		
**7**								
**8**								
**9**	1							
**10**			1	1				
**11**	1						1	
**12**				1				
**13**			1	1				
**14**			1	1				
**15**	1						1	
**16**								
**17**								

Figure S1. Enrichment of the isolated myosin and actin

SDS-gel electophoresis of 2 myosin (left) and 3 actin isolations from muscle samples of the present study showing the enrichment of the 2 proteins. The left lane is a molecular weight ladder.

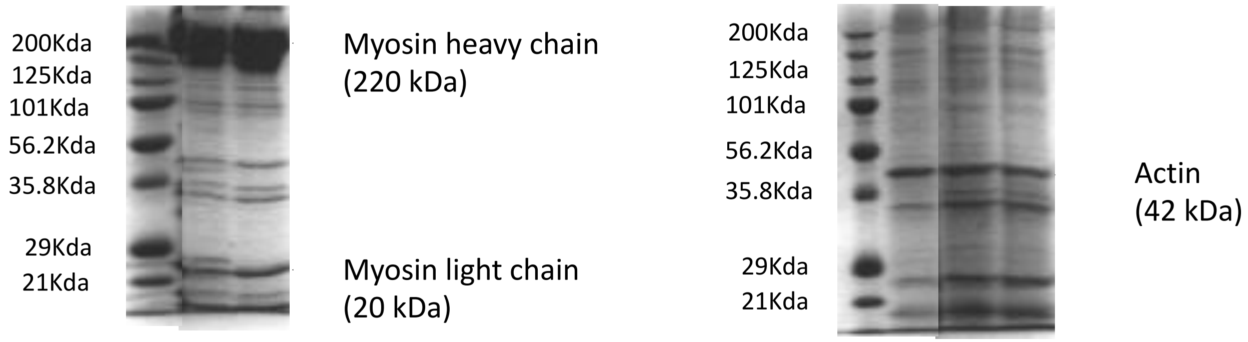

Supplement: Supplementary file 1 [file nutrients-14-03733-s001.zip › nutrients-1843587-supplementary.pdf]
